# Lightweight Chain-Typed Magnetic Fe_3_O_4_@rGO Composites with Enhanced Microwave-Absorption Properties

**DOI:** 10.3390/nano12203699

**Published:** 2022-10-21

**Authors:** Congyi Qian, Xiaohui Liang, Mei Wu, Xingxin Zhang

**Affiliations:** 1 Hangzhou Dianzi University, Hangzhou 310018, China; 2 China Huanqiu Contracting & Engineering Co. (HeBei), Zhuozhou 072750, China

**Keywords:** magnetic composites, microwave absorption, electromagnetic loss, lightweight

## Abstract

A lightweight microwave-absorbing material with a strong electromagnetic-absorption capability of practical significance in the field of electromagnetic compatibility was obtained by adjusting the ratio of Fe_3_O_4_ and rGO. A nanoparticle material with a chain-typed structure consisting of a combination of Fe_3_O_4_ and rGO was produced by a hydrothermal method under an applied magnetic field. The electromagnetic loss property of the Fe_3_O_4_@rGO composites is studied in the frequency range from 2 to 18 GHz. In addition, the reflection loss and the mechanism of microwave absorption are explored. By changing the amounts of rGO, the electromagnetic loss of the Fe_3_O_4_@rGO composites can be effectively regulated, which obtain better reflection loss. The minimum reflection loss of the Fe_3_O_4_@rGO composites is −49.4 dB at 16.2 GHz only with a thickness of 1.75 mm. Thus, the Fe_3_O_4_@rGO composites have an extremely thin thickness and a strong electromagnetic wave absorption capacity, which is a candidate for the development of lightweight magnetic absorbing materials.

## 1. Introduction

With the rapid increase in the number of various electronic devices for different purposes, electromagnetic pollution is detrimental to the correct functioning of various electronic devices and personal safety [1,2,3]. Therefore, lightweight and thin absorbing materials with excellent electromagnetic-absorption performance for electromagnetic compatibility fields are in wide demand. Microwave-absorption materials are capable of converting the energy of electromagnetic waves into alternative forms of energy such as heat [4,5]. Mainly, according to the microwave-absorption machinery, electromagnetic wave absorption materials can be categorized into two major groupings: dielectric-loss materials and magnetic-loss materials [6]. Nonetheless, single-component microwave-absorption materials cannot bring high dielectric and magnetic loss synchronously. Because of the tunable mechanism of dielectric and magnetic loss [7], the integration of magnetic and carbon-based materials is one of the hot spots in the field of microwave absorption [8,9,10]. In particular, the requirement is to acquire materials with strong microwave-absorption reflection loss, wideband effectively absorption bandwidth and thin thickness [11,12].

Recently, a tremendous variety of metallic compounds have obtained adequate research owing to their physical and chemical properties in microwave absorption [13,14]. For example, Green et al. demonstrated that Co_2_P nanoparticles have excellent electromagnetic wave absorption performance with a maximum reflection loss (*R_L_*) of −53.93 dB at a thickness of 2.32 mm [6]. Younes et al. studied carbon nanostructures (CNS)-based mats, whose shielding performance of CNS-Fe_2_O_3_ is 60.29 dB [15]. Zhao et al. studied Ni@CNTs (carbon nanotubes), which exhibit excellent mechanical properties [16]. Tao et al. reported a porous plasmonic Cu_9_S_5_ nanoweb structure with an outstanding microwave absorption, a reflection loss of −55.03 dB, and a maximum functional bandwidth of 4.15 GHz [17]. In addition, due to its excellent magnetic-loss capability, Fe_3_O_4_ is considered to be a high-quality microwave-absorbing material [18,19,20]. For instance, Pan et al. prepared Fe_3_O_4_ nanoribbons by electrostatic spinning and a two-step heat treatment, and the maximum reflection loss of the obtained Fe_3_O_4_ nanoribbons reached −53.93 dB at 2.32 mm thickness [21]. Su et al. constructed the Fe_3_O_4_@C superparticles with a superlattice structure through the organic phase method and the Fe_3_O_4_@C superparticles showed outstanding microwave-absorption performance [22].

Although Fe_3_O_4_ exhibits superior magnetic-loss performance [23], the pure Fe_3_O_4_ cannot show better microwave-absorption performance owing to its poor dielectric properties [24]. Hence, it is usually combined with other lightweight materials with better dielectric properties to obtain excellent microwave-absorbing materials [25]. For example, Choi et al. developed Fe_3_O_4_/MWCNTs (multi-walled carbon nanotubes), enhancing the electromagnetic shielding of the X band [26]. Bai et al. fabricated rGO (reduced graphene oxide)/Fe_3_O_4_ composites with −72.7 dB at 4.5 mm [27]. Sun et al. obtained the Fe_3_O_4_/rGO/PANI (polyaniline)-paraffine composite with −45.0 dB at 8.5 GHz and the effective absorbing bandwidth is 12.2 GHz [28]. Among them, graphene, on account of its special two-dimensional structure and magnetic and dielectric properties [29], has been widely investigated [8,30]. For instance, Wang et al. constructed flower-like CoFe2O4@graphene by the method of spray-drying route and the maximum reflection loss reached −42 dB [31]. Zhang et al. fabricated three-dimensional porous polyurethane (PU) and reduced graphene oxide (rGO) foams and they discussed the possible microwave-absorption mechanisms. For the PU/rGO foam, the maximum *R_L_* value of 16.55 GHz can reach −46.2 dB with a thickness of 2 mm [32]. Wang et al. fabricated a Ni/rGO/EP (epoxy) composite foam with a maximum reflection loss of even −58.23 dB at 8.4 GHz and a thickness of 2.5 mm [33]. In conclusion, because of its light weight and high dielectric loss, rGO became a significant component combined with other materials to be a microwave absorber [30,34,35].

In this study, a novel chain-typed Fe_3_O_4_@rGO nanoparticle material was manufactured by the hydrothermal method with a magnetic field applied, which shows better electromagnetic microwave absorption. The reflection loss of Fe_3_O_4_@rGO nanobelt materials with various thicknesses over the whole frequency range from 2–18 GHz was researched, and the microwave-absorption mechanism was investigated. In the process of practice, we found that due to impedance matching, dielectric loss and magnetic loss, the reflection loss is the largest and the absorption performance is the best when the thickness is 1.75 mm. Typically, the minimum refection loss of the Fe_3_O_4_@rGO nanoparticles was identified to reach −49.4 dB at 16.2 GHz with a thickness of 1.75 mm.

## 2. Materials and Methods

### 2.1. Materials

Commercially available Ethylene glycol (EG, purity: 99.0%), Iron (III) chloride hexahydrate (FeCl_3_·6H_2_O, purity: 99.0%), and Sodium acetate trihydrate (CH_3_COONa·3H_2_O, purity: 99.5%) was obtained from Aladdin Bio-Chem Technology Co., Ltd., (Shanghai, China). Graphene oxide (GO) was purchased by Xianfeng Nano Technology Co., Ltd., (Nanjing, China).

### 2.2. Fabrication of Fe_3_O_4_@rGO

**Figure 1:**
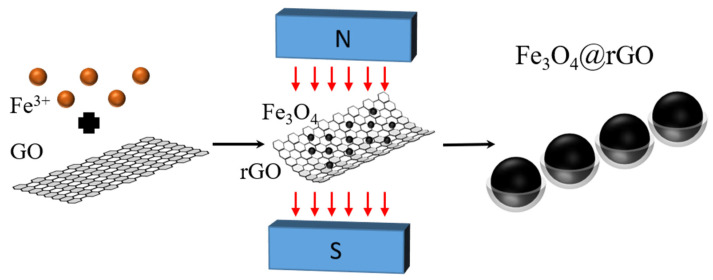
Schematic diagram of the synthesis of chain-typed Fe_3_O_4_@rGO nanoparticles by hydrothermal method in the presence with an applied magnetic field.

The synthesis process of chain-typed Fe_3_O_4_@rGO composite nanoparticles is schematically shown in Figure 1. We dissolved 1.62 g FeCl_3_·6H_2_O and an amount of GO with a concentration of 2 mg/mL in 40 mL of ethylene glycol solution and mix ultrasonically for 20 min. In addition, we dissolved 3 g sodium acetate in 60 mL of ethylene glycol and mix ultrasonically for 10 min. Then, the above two solutions were mixed and stirred magnetically for 0.5 h. After that, they were transferred to a 100 mL reaction vessel with magnets on both sides of the vessel where the magnetic chains are oriented along a specific direction, which causes the ferromagnetic Fe_3_O_4_ nanoparticles to align along that direction, forming a one-dimensional chain-typed structure. Then they were heated at 180 °C for 18 h to obtain a black precipitate. The black precipitate was washed by centrifugal filtration with distilled water and anhydrous ethanol, and finally freeze-dried to obtain chain-typed Fe_3_O_4_@rGO composites. The Fe_3_O_4_@rGO composite nanoparticles constructed with 6 mL, 8 mL, and 10 mL of GO were tagged as S1, S2, and S3, respectively. S1, S2, and S3 are samples of the magnetic Fe_3_O_4_ nanoparticles surrounded by increased numbers of rGO layers. GO addition, sample diameter, and saturation magnetization intensity of samples S1, S2, and S3 are presented in Table 1. It can be seen from Table 1 that the dimensions of S1, S2, and S3 are close, but the saturation magnetization intensity of the samples decreases with the increase in the GO amount.

### 2.3. Characterization and Measurement

The lattice characteristics of the Fe_3_O_4_@rGO nanocomposites were probed using an X-ray powder diffractometer (XRD) (Rigku, Kyocera, Japan) with CuK_α_ radiation at a wavelength of 1.5418 Å. The elemental and valence bond information of the nanocomposites was obtained using X-ray photoelectron spectroscopy (XPS) of PHI 5000 Versa Probe (Physical Electronics, Shanghai, China). Hysteresis lines of the Fe_3_O_4_@rGO nanocomposites were measured with a Lakeshore 7400 series vibrating sample magnetometer (Lakeshore Cryotronics, Westerville, OH, USA). Test samples were obtained by homogeneously admixing 40% paraffin wax with 60% Fe_3_O_4_@rGO nanoparticles compressed into an annulus of 1.52 mm inner circle radius and 3.5 mm outer circle radius. The complex permittivity and complex permeability of the tested samples were then obtained by the Agilent PNA N5224A vector network analyzer (Agilent Technologies, Santa Clara, CA, USA) with the coaxial line method over a frequency range from 2–18 GHz.

## 3. Results and Discussion

**Figure 2:**
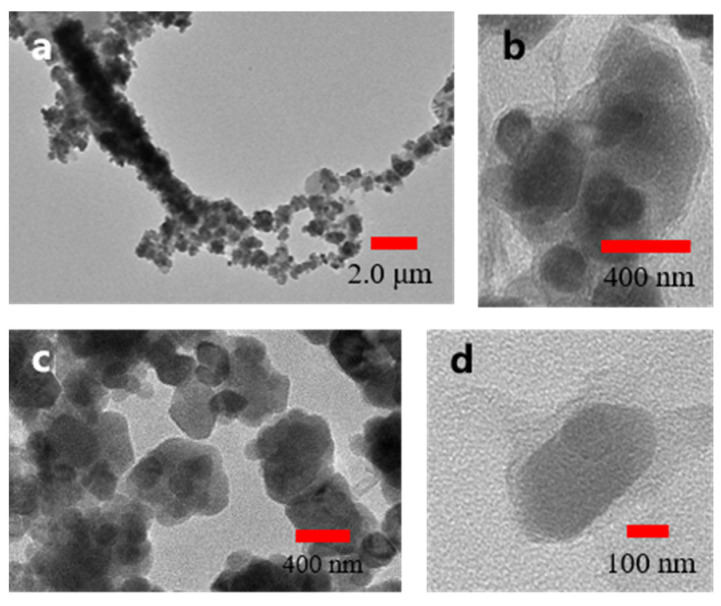
Transmission electron microscopy images of Fe_3_O_4_@rGO nanoparticles at variable magnifications (**a**–**d**).

Figure 2 presents the TEM images, in which the structure and characterization of the Fe_3_O_4_@rGO nanoparticles are shown. As shown in Figure 2a, Fe_3_O_4_@rGO nanoparticle individuals form a chain structure under the action of a magnetic field. Figure 2a demonstrated that, due to the application of an external magnetic field in the synthesis process, the overall arrangement of Fe_3_O_4_@rGO nanoparticles has a certain orientation, forming a resemble one-dimensional structure. The chain structure is beneficial to microwave absorption. From Figure 2b,c, the Fe_3_O_4_ nanoparticles are wrapped by rGO. The Fe_3_O_4_ nanoparticles were coated with multiple layers of rGO, obtaining a cluster of interconnected structures. Furthermore, as seen in Figure 2c,d, a single Fe_3_O_4_@rGO nanoparticle has an average diameter range from 150 nm to 450 nm. The single Fe_3_O_4_@rGO is formed by rGO wrapped around the Fe_3_O_4_ particles with a diameter of about 380 nm.

**Figure 3:**
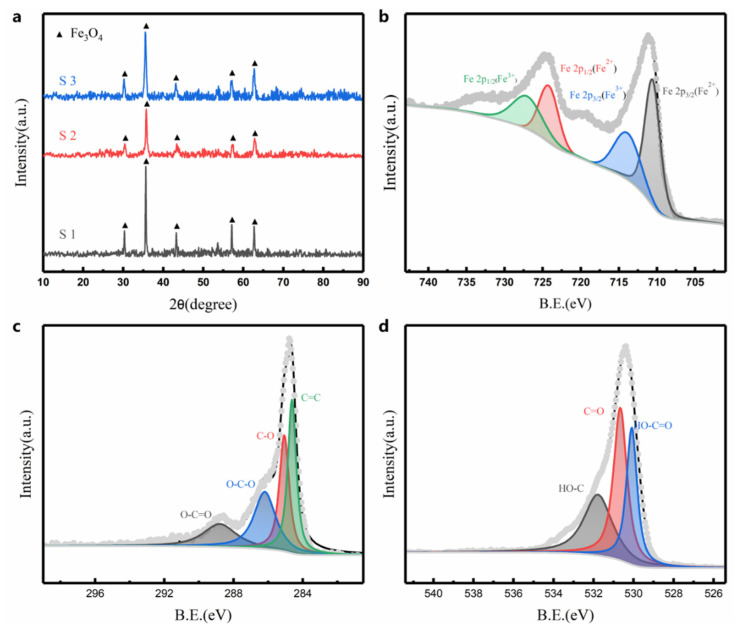
(**a**) XRD patterns of annealed Fe_3_O_4_@rGO. High-resolution XPS spectra of (**b**) Fe 2p, (**c**) C 1s and (**d**) O 1s.

The XRD pattern of Fe_3_O_4_@rGO composites is shown Figure 3a. The diffraction peaks of all samples belong to the cubic Fe_3_O_4_ phase (according to JCPDS card No. 79-0419), demonstrating the high degree of precision of the Fe_3_O_4_ phase in the sample material. To further investigate the elemental and valence bonding properties of the Fe_3_O_4_@rGO composites, XPS tests were carried out. The two characteristic peaks of the Fe 2p spectrum (Figure 3b) (Fe 2p1/2 at 711.2 eV and Fe 2p3/2 at 724.6 eV) were decomposed into two Fe^2+^ peaks (710.7 and 724.4 eV) and two Fe^3+^ peaks (714.2 and 727.4 eV) [36]. This evidence demonstrated the high degree of precision of the Fe_3_O_4_ phase. From the C 1s spectrum (Figure 3c), it is clear that it can be decomposed into four peaks with peak binding energies (B.E.) of 284.6 eV, 285.1 eV, 286.2 eV and 288.8 eV, which belong to O-C=O, O-C-O, C-O and C=C, respectively. The O 1s spectrum (Figure 3d) can be separated into three peaks with peak binding energies of 530.1 eV, 530.7 eV and 530.1 eV, ascribed to HO-C=O, C=O and O-C=O, respectively. The decrease in the intensity of O 1s in the composite compared to GO indicates that GO is effectively reduced, proving the presence of rGO in the resulting material.

The *R_L_* of the Fe_3_O_4_@rGO composites reveals the final electromagnetic properties, based on the transmission line theory [37]. *R_L_* can be derived from *ε_r_* and *µ_r_* through the following equation [38]:

(1) $$Z_{in}=Z_{0}{\left(\mu_{r}/\varepsilon_{r}\right)}^{1/2}\tan\;h\left[j\left(2\pi fd/c\right)\;{\left(\varepsilon_{r}\mu_{r}\right)}^{1/2}\right]$$

(2) $$R_{L}=20\log\left(Z_{in}-Z_{0}\right)/\left(Z_{in}+Z_{0}\right)$$

where *Z*_0_ represents the wave impedance in free space, *c* represents the velocity of light, *d* represents the thickness of the absorbing material, *f* represents the frequency of the electromagnetic wave, and *Z_in_* represents the input impedance of the absorbing material.

According to the above formula, it can be seen that *R_L_* can be greatly affected by permittivity and permeability. In this study, the change of the permittivity, permeability and the exploration of the maximum reflection loss is realized by adjusting the amount of the rGO contents. The specific methods for adjusting the magnetic permeability and dielectric permittivity are described in detail later.

**Figure 4:**
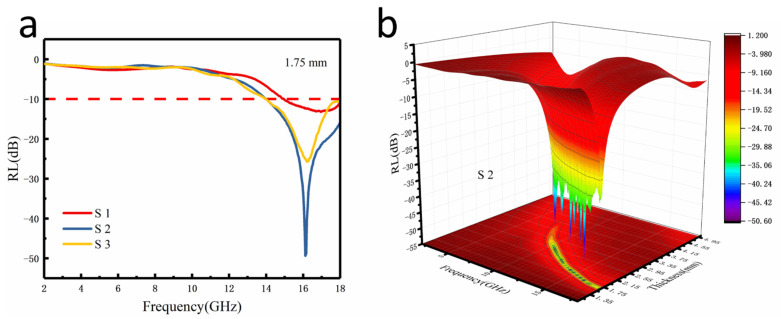
(**a**) Frequency-dependent reflection loss curves of S1, S2, and S3. (**b**) 3D representation of reflection-loss values of S2.

In practical applications, the reflection loss is required to be less than −10 dB, which is an indication that more than 90% of the incident microwaves are available for absorption. As can be seen from Figure 4a, the frequency band of the reflection-loss curve under the marked −10 dB red dashed line has effective absorption performance. Figure 4a shows the reflection loss of the Fe_3_O_4_@rGO composites with a thickness of 1.75 mm. For S1, the minimum reflection loss is −13.2 dB at 16.7 GHz, and the effective bandwidth is 3.4 GHz. For S2, the minimum reflection loss is −49.4 dB at 16.2 GHz, and the effective bandwidth is 3.96 GHz. For S3, the minimum reflection loss is −25.7 dB at 16.2 GHz, and the effective bandwidth is 4.04 GHz. It can be seen from Figure 4a that sample S2 has a higher microwave-absorption capacity and a wider absorption-frequency bandwidth with a thinner thickness compared to the remaining two samples. As can be seen in Figure 4b and Figure S1, both the S1 and S2 samples can effectively absorb electromagnetic waves (*R_L_* < −10 dB) from the thickness of 1.5 mm to 5 mm. As can be seen from Figure S2, the S3 sample can absorb electromagnetic waves with a thickness of 1.45 mm to 5 mm. Combined with Figure S3, the pure rGO can been seen to have a reflection loss >−10 dB in any frequency band and thickness, which cannot absorb microwaves effectively, but the combination with the Fe_3_O_4_ particles under the condition of the applied magnetic field makes the material have a better wave absorption performance. In brief, the Fe_3_O_4_@rGO nanoparticles are lightweight materials that can effectively absorb electromagnetic waves at a very thin thickness, and they have a strong microwave-absorption capacity with a wide frequency range.

**Figure 5:**
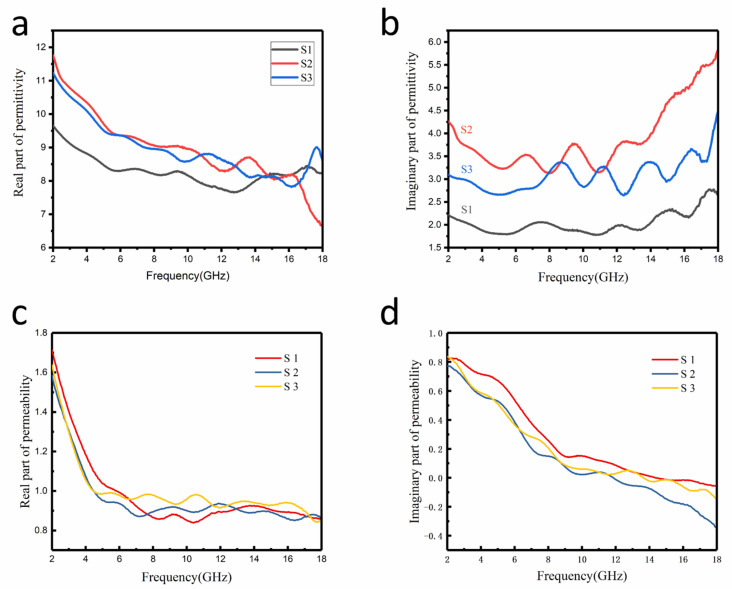
(**a**) Real part of the permittivity. (**b**) The imaginary part of the permittivity. (**c**) Real part of permeability. (**d**) The imaginary part of permeability.

**Figure 6:**
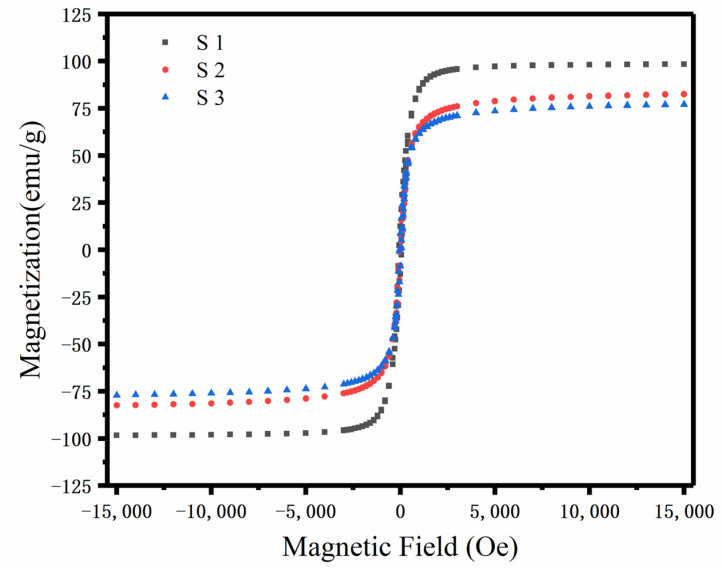
Magnetic field-dependent magnetization curves of S1, S2, and S3.

Overall, the microwave-absorption performance clearly depends on the relative complex permittivity (*ε_r_ = ε′ − jε″*) and the magnetic permeability (*µ_r_ = µ′ − jµ″*). The real part of the electromagnetic parameters (*ε′* and *µ′*) reflects the storage capacity of the electromagnetic energy. Correspondingly, the imaginary part of the electromagnetic parameters represents the loss of the electromagnetic energy. It is necessary to analyze the relative complex permittivity and permeability to grope for the microwave-absorption mechanisms [39]. As can be seen in Figure 5a,b, the complex permittivity of the Fe_3_O_4_@rGO composites dramatically increased with the addition of rGO, and the rGO improved the electromagnetic storage and electromagnetic wave loss ability. Thus, the permittivity of the Fe_3_O_4_@rGO composites relies on the addition of rGO. In Figure 5b, the imaginary part of the dielectric constant increases with the addition of rGO, which means the dielectric loss has been enhanced. Meanwhile, at the frequency range from 2–18 GHz, the dielectric loss primarily relies on the polarization relaxation loss and conductivity losses [40]. Because the conductivity losses are only valid in a homogeneous system, the dielectric loss of the Fe_3_O_4_@rGO composites mainly comes from the polarization relaxation loss occurring in the interfaces of Fe_3_O_4_ and rGO. As seen in Figure S4, rGO has a very large complex dielectric permittivity, but the complex permeability is lower. The dielectric permittivity of the Fe_3_O_4_@rGO composites is enhanced by the addition of rGO, which improves the dielectric loss of the composites. In addition, polarization relaxation generally depends on dipole orientation polarization and interfacial polarization. As shown in Figure 5,b, the polarization has occurred in the 6–18 GHz, and this is because the introduction of rGO can enhance the interfacial polarization ability of the composites. In addition, defects in the multilayer rGO produce dipole polarization. Thus, the rGO enhanced the ability of interfacial and dipole polarization of the Fe_3_O_4_@rGO composites, which also improved dielectric loss of the composites. Figure 5c,d exhibit that the Fe_3_O_4_@rGO composites perform high permeability due to the addition of the Fe_3_O_4_ nanoparticles. From Figure 5c,d, it can be seen there are several resonance peaks and the magnetic permeability slowly decreases with the increase in rGO and the magnetic loss slightly decreases. The magnetic losses over the whole frequency range from 2–18 GHz are mainly caused by the natural resonance modes. The natural resonance frequency has the relation *f_r_* = *γH_a_*/2*π*, in which the *H_a_* is the anisotropic field, *g* is the Lande factor and *γ* is the gyromagnetic ration [41]. As we all know, the multiple resonances can be obtained due to the multiple *g* factors that originate from the Fe^3+^ ions and exchange coupling among Fe^3+^ and Fe^2+^ in the Fe_3_O_4_ nanoparticles [42]. Therefore, there are natural resonance in the Fe_3_O_4_@rGO composites. Furthermore, Figure 6 exhibited that the saturation magnetizations (*M_s_*) of S1, S2, and S3 are 98.39, 82.47, and 77.09 emu g^−1^, respectively. It can be seen that the saturation magnetization intensity decreases as the amount of rGO increases. These may be attributed to the fact that with the increase in the relative content of rGO, the Fe_3_O_4_ nanoparticles were wrapped in more layers of rGO, and the rGO makes magnetism of the Fe_3_O_4_@rGO composites gradually lower. Therefore, by regulating the relative amount of rGO, the magnetic properties of the Fe_3_O_4_@rGO nanoparticles can be changed. In addition, the hysteresis loop covers a small area, so the Fe_3_O_4_@rGO composites have a small hysteresis loss.

The dielectric loss angle tangent ($\tan\delta_{e}$) and the magnetic loss angle tangent ($\tan\delta_{m}$) can reflect the dielectric loss and magnetic loss per unit volume of the material, respectively, and they are calculated by the following equations:

(3) $$\tan\delta_{e}=\frac{\varepsilon^{\prime \prime }}{\varepsilon^{\prime }}$$

(4) $$\tan\delta_{m}=\frac{\mu^{\prime \prime }}{\mu^{\prime }}$$

**Figure 7:**
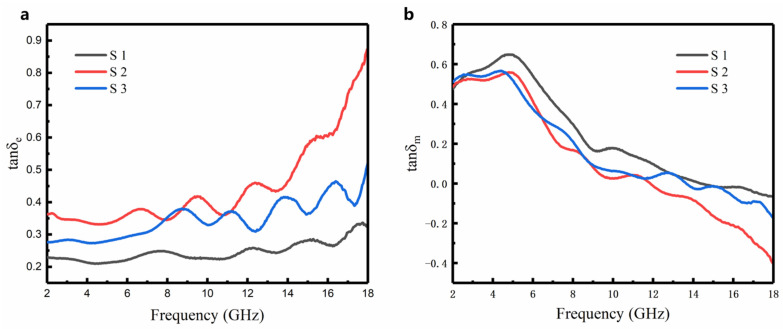
(**a**) the dielectric loss tan *δ_e_*, (**b**) the magnetic loss tan *δ_m_*.

**Figure 8:**
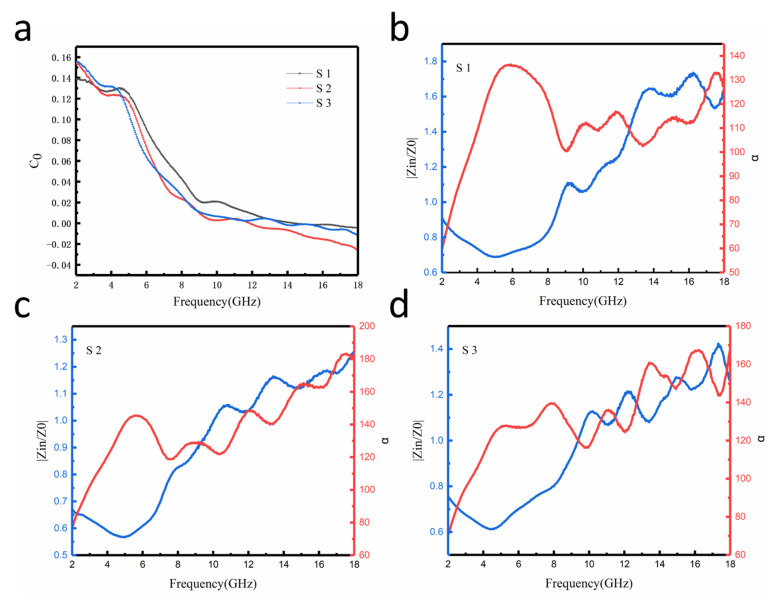
(**a**) Eddy current curve of the Fe_3_O_4_@rGO composites. Impedance matching and loss curves of (**b**) S1, (**c**) S2, and (**d**) S3 for a thickness of 1.75 mm.

Figure 7a shows the dielectric loss of the Fe_3_O_4_@rGO nanoparticles with various components. The tan *δ_e_* of S2 increases rapidly with the increase in frequency, which is due to the interfacial relaxation loss caused by charge accumulation on the non-uniform surface of the Fe_3_O_4_ nanoparticles and rGO [43]. Figure 8b shows that the magnetic loss of each component sample increases first and then decreases with the increase in frequency. Natural resonance mainly impacts the low frequency band, and exchange resonance mainly affects the high-frequency band [44]. In addition, by adding magnets during the synthesis process, the magnetic chains are oriented along a specific direction, which causes the ferromagnetic Fe_3_O_4_ nanoparticles to align along that direction, forming a one-dimensional chain-typed structure with increased complex permeability and enhanced wave absorption performance. Therefore, all three samples exhibit relatively good magnetic loss [45]. It can be seen from Figure 7 that the dielectric loss is stronger than the magnetic loss throughout the high-frequency band. It is because the unique anisotropy in the one-dimensional structure of the Fe_3_O_4_@rGO nanoparticles increases its conductive ability and its dielectric loss. The complex permeability of the three sets of samples is relatively flat in the frequency range from 2–18 GHz. However, the magnetic loss appears as a natural resonance peak around 4.8 GHz. It can be inferred that the exchange formant is higher than 18 GHz, because when the particle size of the material decreases to the order of a nanometer, both the natural and exchange formants move to the high-frequency band [46].

As we all know, the magnetic loss includes the domain wall resonance, the hysteresis loss, the eddy current effect, and the natural resonance [47]. The domain wall resonances produced by the multi-domain materials exist exclusively in the lower frequency range ( < 0.1 GHz) [48]; thus, there is no domain wall in the 2–18 GHz frequency. The eddy current loss will hinder the electromagnetic wave from entering inside the absorbing material, resulting in the absorption performance decline. However, for most absorbers, the eddy current losses are difficult to control. The eddy current losses are computed by the formula below [49]:

(5) $$C_{0}=\mu^{\prime \prime }{\left(\mu^{\prime }\right)}^{-2}f{\;}^{-1}=\frac{2}{3}\pi \mu_{0}\sigma d^{2}$$

where *σ* stands for electrical conductivity and *d* stands for the thickness of materials. From the above formula, if the magnetic loss primarily depends on the eddy current loss, the *C*_0_ will be a constant. It is seen in Figure 8a that the *C*_0_ values of the Fe_3_O_4_@rGO composites are not a constant. Therefore, eddy current loss is not the main cause of the magnetic loss in the Fe_3_O_4_@rGO composites. Combined with Figure 6, the magnetic loss is caused by low coercivity and high-saturation magnetization.

In addition to dielectric and magnetic losses, the electromagnetic-absorption capacity is also related to the electromagnetic impedance matching. If the input impedance (*Z_in_*) and the characteristic impedance (*Z*_0_) are almost equal, the percentage of electromagnetic waves entering the material is higher. Figure 8b–d show the impedance matching ($\left|Z_{in}/Z_{0}\right|$) curve of S1, S2, and S3 and the variation curve of electromagnetic attenuation capacity concerning frequency, respectively. The electromagnetic attenuation constant (α) can be computed by the formula below [50]:

(6) $$\alpha =\frac{\sqrt{2}\pi f}{c}\sqrt{\left(\mu^{\prime \prime }\varepsilon^{\prime \prime }-\mu^{\prime }\varepsilon^{\prime }\right)+\sqrt{{\left(\mu^{\prime \prime }\varepsilon^{\prime \prime }-\mu^{\prime }\varepsilon^{\prime }\right)}^{2}+{\left(\varepsilon^{\prime }\mu^{\prime \prime }+\varepsilon^{\prime \prime }\mu^{\prime }\right)}^{2}}}$$

more excellent impedance matching means less reflected waves [51], and a larger electromagnetic attenuation constant means that the electromagnetic waves entering the material can be fully absorbed [52]. These two parameters determine the absorbing capacity. Figure 8b–d exhibit that compared with the other two samples, S2 has higher electromagnetic losses at all frequencies. Moreover, at 16.2 GHz, S2 has a better electromagnetic impedance match ($\left|Z_{in}/Z_{0}\right|\approx 1$). At this point, the absorption of electromagnetic waves by the Fe_3_O_4_@rGO nanoparticles is the highest. Therefore, although the dielectric loss of the S3 samples is stronger than that of the S2 samples in some frequency bands as the rGO increases, accordingly, the impedance matching of the S3 samples becomes poorer and the proportion of reflections in the incident electromagnetic waves increases, resulting in a poorer overall wave absorption performance of the material. Therefore, the reflection loss of S2 obtains a maximum of 16.2 GHz, which is consist with Figure 4a.

When the impedance matching is good ($\left|Z_{in}/Z_{0}\right|=1$), all the electromagnetic waves go into the absorbing material [53]. When the impedance matching is poor, the proportion of the reflected part of the incident electromagnetic wave increases and the material reflection loss decreases. The electromagnetic parameters of absorbing materials make them have inherent magnetic and dielectric losses. A portion of the electromagnetic waves entering the material is absorbed and transformed into heat and some other energy types [54]. Of course, there is still a tiny fraction of microwaves transmitted into the absorbing material; in practical applications, we want this part of the electromagnetic wave as little as possible.

In this study, because the Fe_3_O_4_ nanoparticles are coated with highly conductive rGO and form a one-dimensional structure in the presence of a magnetic field, the Fe_3_O_4_@rGO nanoparticles exhibit good electrical conductivity [55]. High electrical conductivity brings high dielectric loss for the absorbing materials [54]. The Fe_3_O_4_ nanoparticles have strong magnetic properties that enable the Fe_3_O_4_@rGO nanoparticles to have an excellent reflection loss with a thin thickness. Therefore, the thin thickness of the material makes the Fe_3_O_4_@rGO composites become lightweight absorbing materials with excellent performance.

## 4. Conclusions

In conclusion, the chain-typed Fe_3_O_4_@rGO composites are obtained by the hydrothermal method with an applied magnetic field. Due to the addition of the applied magnetic field, the Fe_3_O_4_ magnetic nanoparticles are aligned along the magnetic field line direction and wrapped by rGO to form a one-dimensional structure, which makes the magnetic loss increase and the reflection loss enhance. By adjusting the amounts of rGO, the permeability and dielectric constant of the Fe_3_O_4_@rGO composites can be changed in a more controlled manner, resulting in the electromagnetic-absorption capacity of the material changed. With the increase in rGO, the dielectric constant increases in a certain frequency band, which makes the dielectric loss increase, leading to the increase in the reflection ratio in the incident wave. Thus, because of the better dielectric loss, magnetic loss and impedance matching, the maximum reflection loss of the S2 sample is obtained when the GO addition is 8 mL. The reflection loss of the Fe_3_O_4_@rGO composites is minimum −49.4 dB at 16.2 GHz with a thickness of 1.75 mm. Finally, a relatively thin and superior absorbing material is acquired, which has a potential practical significance in lightweight absorption.

## Figures and Tables

**Table 1:** GO addition, sample diameter, and saturation magnetization intensity of samples S1, S2, and S3.

Materials	GO (mL)	Diameter (nm)	Magnetization (emu g^−1^)
S1	6	375	98.39
S2	8	392	82.47
S3	10	386	77.09

## Data Availability

The data that support the findings of this study are available from the corresponding authors, upon reasonable request.

## Supplementary Materials

The following supporting information can be downloaded at: https://www.mdpi.com/article/10.3390/nano12203699/s1. The 3D representation of reflection-loss values of the Fe_3_O_4_@rGO composites (Figures S1 and S2), the 3D representation of reflection-loss values of rGO (Figure S3) and complex permeability and complex dielectric permittivity of rGO (Figure S4).
