# Supplementary material for: Lightweight Chain-Typed Magnetic Fe3O4@rGO Composites with Enhanced Microwave-Absorption Properties
Source: Nanomaterials (Basel). 2022 Oct 21;12(20):3699. doi: 10.3390/nano12203699 (PMC9612295; doi:10.3390/nano12203699)
Supplement: Supplementary file 1 [file nanomaterials-12-03699-s001.zip › nanomaterials-1953262-supplementary.pdf]

nanomaterials

MDPI

Supplementary Materials

Lightweight Chain-Typed Magnetic Fe_3O_4@rGO Composites with Enhanced Microwave-Absorption Properties

Congyi Qian [1], Xiaohui Liang [1,*], Mei Wu [1] and Xingxin Zhang [2]

[1] Hangzhou Dianzi University, Hangzhou 310018, China
[2] China Huanqiu Contracting & Engineering Co. (HeBei), Zhuozhou 072750, China
* Correspondence: xhliang@hdu.edu.cn

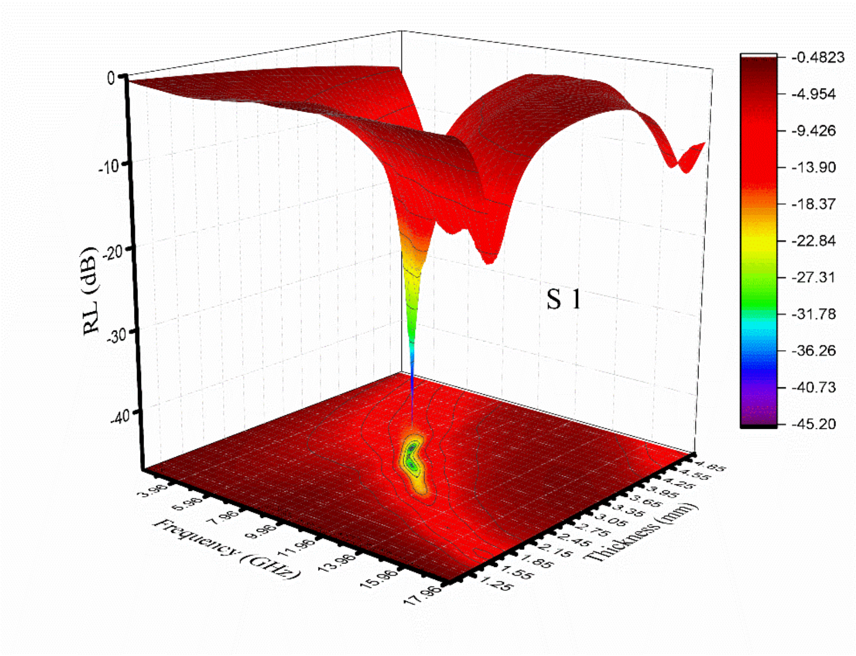

Figure S1. 3 D representation of reflection loss values of S1.

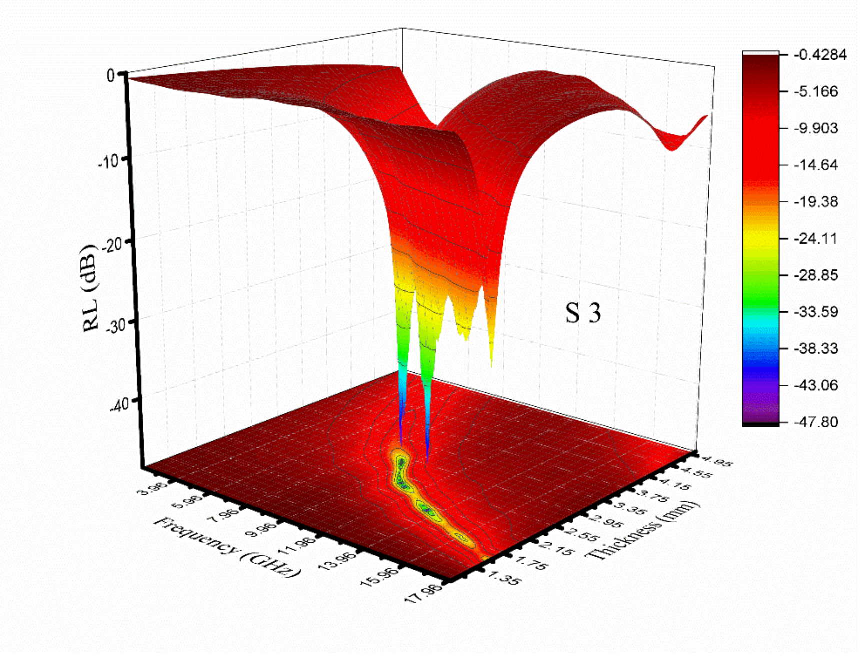

Figure S2. 3 D representation of reflection loss values of S3.

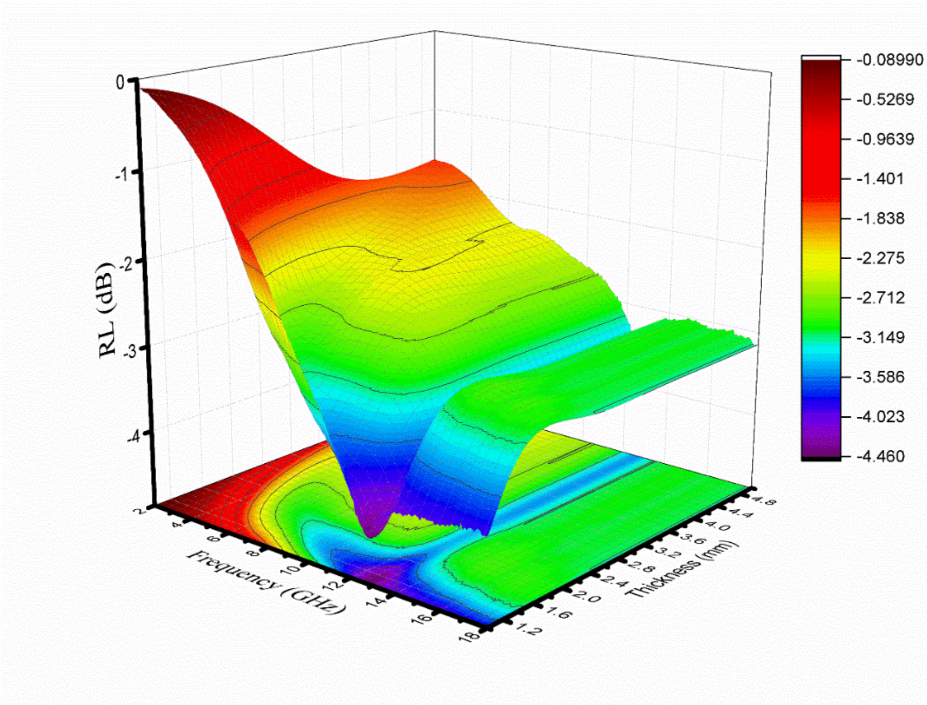

Figure S3. 3 D representation of reflection loss values of rGO.

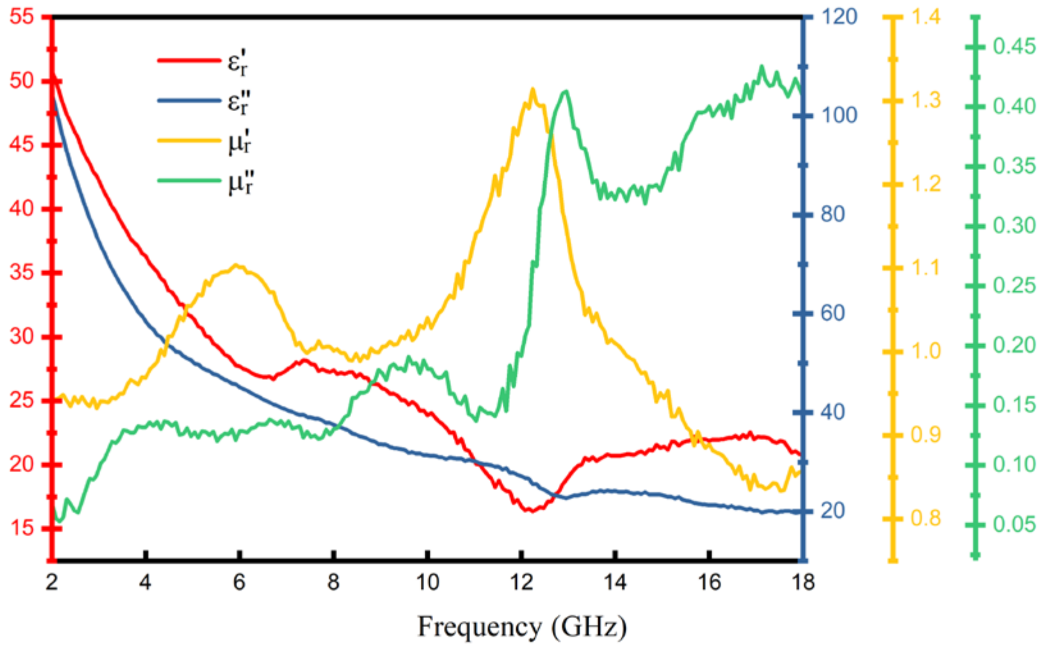

Figure S4. Complex permeability and complex dielectric permittivity of rGO.
